# False-negative factors of percutaneous transluminal clamp biopsy for suspected malignant biliary stricture: 194 cases analyzed from a single center

**DOI:** 10.1186/s13244-024-01675-y

**Published:** 2024-04-12

**Authors:** Chengzhi Zhang, Yipu Li, Mengyao Song, Zhanguo Sun, Xinwei Han, Jianzhuang Ren, Dechao Jiao

**Affiliations:** Department of Interventional Radiology, The First Affiliated Hospital of Zhengzhou University, Interventional Therapy Institute of Zhengzhou University, No. 1 Jianshe East Road, Zhengzhou City, 450000 Henan Province China

**Keywords:** Biliary stricture, Cholangiocarcinoma, Cholangiography, Clamp biopsy, Interventional angiography

## Abstract

**Objective:**

To study the predictive factors of false negatives in the diagnosis of biliary stricture (BS) by percutaneous transluminal clamp biopsy (PTCB).

**Method:**

From January 2016 to January 2021, 194 patients with a high suspicion of malignant tumors due to BS underwent PTCB during biliary drainage at our department. The final diagnosis was confirmed by postoperative pathology, other tissue or cell evidence, or medical imaging follow-up. Univariate and multivariate regression analyses were performed on the pathological results, summarizing the independent risk factors for false-negative value (FNV) to help further clinical diagnosis and treatment.

**Results:**

Of the 194 cases, 176 and 18 cases were finally diagnosed as malignant and benign BS, respectively, compared to 144 and 50 cases by PTCB, including 32 false-negative cases. The sensitivity, specificity, false-positive value, and FNV of PTCB were 81.8%, 100%, 0%, and 18.2%, respectively. Multivariate analysis showed that non-cholangiocarcinoma BS was an independent risk factor for FNV of PTCB (odds ratio 7.5 (95% CI 1.74–32.6), *p* < 0.01).

**Conclusion:**

PTCB is an effective minimally invasive interventional technique for BS diagnosis. Non-cholangiocarcinoma BS is an independent risk factor for FNV.

**Critical relevance statement:**

Identifying factors that are predictive of false-negative results by percutaneous transluminal clamp biopsy in the setting of biliary stricture may have a guiding effect on clinical practice.

**Key points:**

• Factors predictive of false negatives in the diagnosis of biliary stricture etiology by PTCB may aid in the interpretation of results.

• Non-cholangiocarcinoma BS is an independent risk factor for FNV on PTCB.

• PTCB is an effective minimally invasive interventional technique for BS diagnosis.

**Graphical Abstract:**

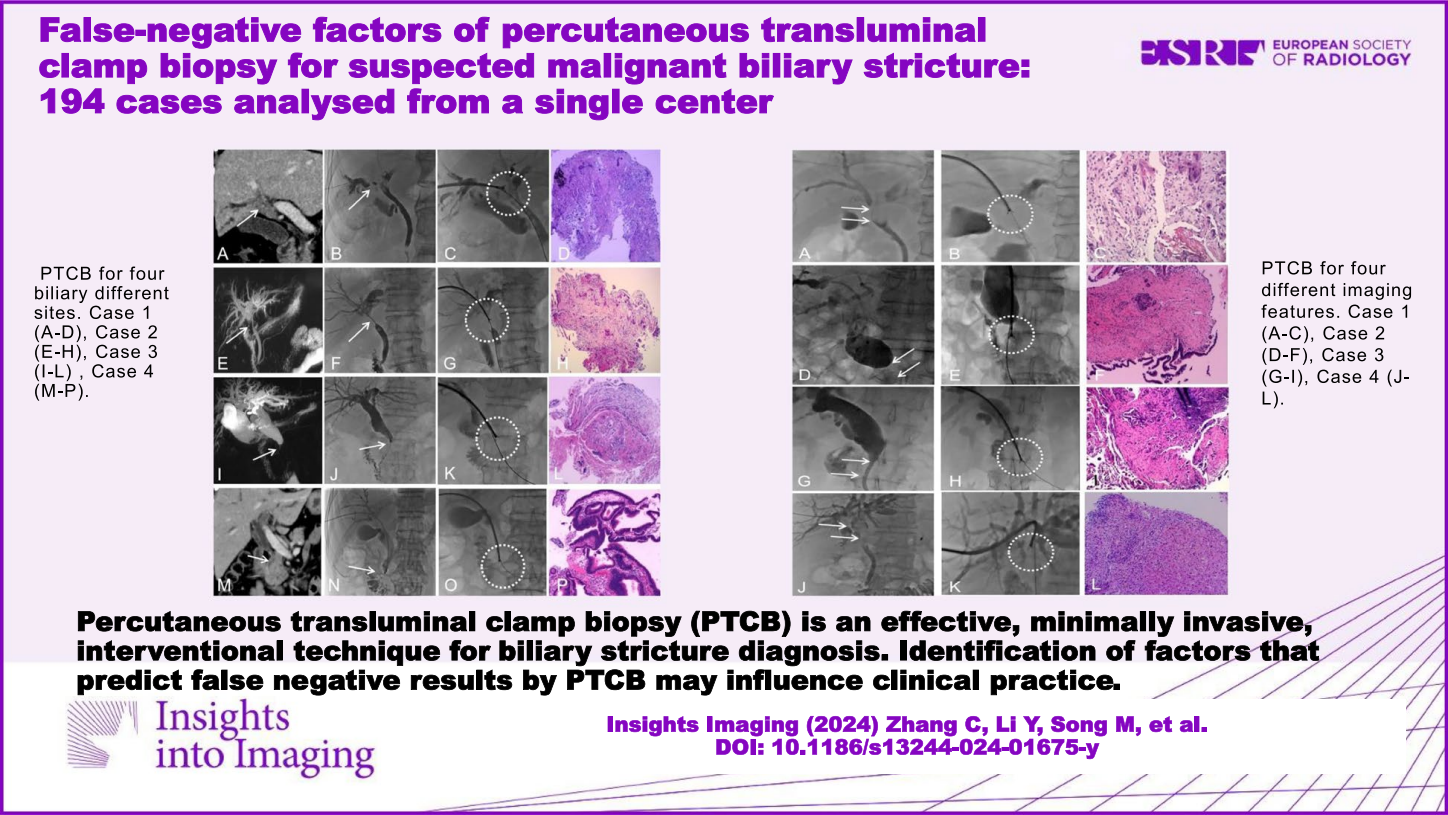

## Introduction

Biliary strictures (BS) are caused by intra- or extrahepatic malignant or benign diseases and result in high biliary pressure above the stricture; they predominantly manifest with signs and symptoms of fatty diarrhea, jaundice, bleeding diatheses, and pancreatitis [1, 2]. When bile ductal pressure increases past a certain threshold, direct bilirubin will reversely flow into the blood system, resulting in pathophysiological changes in multiple organs. Additionally, intestinal bacteria can easily invade the biliary system and develop into bacteremia and toxemia, leading to multisystem organ failure [3]. There are many reasons for mechanical BS, such as malignant tumors, biliary stones, inflammation, and postoperative anastomotic stenosis. In the past few decades, great progress has been made in BS imaging, such as intraluminal ultrasound, magnetic resonance cholangiopancreatography (MRCP), and enhanced CT scanning [4, 5]. These non-invasive imaging systems can quickly obtain relevant anatomical BS information but do not allow for biopsy of the stricture—the most critical information for the final diagnosis.

To obtain intrabiliary sampling, there are currently three methods: (1) percutaneous biopsy/fine needle aspiration under CT/US guidance [6], (2) endoscopic retrograde cholangiopancreatography (ERCP)-guided intrabiliary clamp biopsy/brush inspection [7], and (3) percutaneous transluminal clamp biopsy (PTCB)/brush inspection under fluoroscopic guidance [8]. Percutaneous biopsy has relatively high technical operation requirements and a high risk of puncture-related bleeding and bile extravasation, especially for very small tumors. Additionally, a biliary drainage procedure cannot be performed simultaneously. ERCP-guided biopsy is simple and convenient and is suitable for lower extrahepatic BS; however, ERCP-guided biopsy for hilar BS has relatively low technical success (35.0–53.4% from previous studies [9, 10]). Moreover, procedures requiring ERCP are more expensive than other methods, partially due to the use of general anesthetics. PTCB under local anesthesia is suitable for all positions of the bile duct and allows for simultaneous biliary drainage. Elyaderani and Gabriele first reported PTCB in 1980 [11], and increasing clinical data from the USA, Europe, and China have shown that it is an efficient and safe technology. A 2022 meta-analysis from Jeon et al. [12] analyzed 1762 PTCB procedures from 14 studies and found that the sensitivity and specificity were 80% and 100%, respectively. PTCB is highly sensitive and specific for the diagnosis of malignant biliary strictures. In previous studies of PTCB by Augustin et al. [6] and Oggero et al. [13] false-negative results have been reported; however, the risk factors contributing to false-negative results have not been systematically analyzed. Few studies have focused on the factors influencing false-negative values (FNV) in large sample analyses. It is paramount to understand if there are technical aspects of risk factors that contribute to false negative PTCB. This could be used to guide clinics in improving methodologies and avoiding false-negative results. The purpose of this study was to evaluate the diagnostic accuracy of PTCB among 194 patients with BS and to determine the predictive factors related to FNV.

## Materials and methods

This study was approved by the Ethics Committee of our center. From January 2016 to January 2021, 248 patients with BS underwent PTCB combined with biliary drainage in our department. The inclusion criteria for the study were as follows: (1) age range 18–80 years, (2) direct bilirubin (DB) was at least three times higher than the normal value, (3) clinical manifestations were typical for a BS (e.g., jaundice, cholangitis, and fever), (4) Eastern Cooperative Oncology Group (ECOG) score of 0–2, (5) complete clinical data and more than 6 months of follow-up, and (6) platelet count > 50 × 10^9^/L and prothrombin time (PT) ≤ 25 s. The exclusion criteria were as follows: (1) uncontrollable ascites, (2) severe coagulopathy, and (3) severe cardiopulmonary dysfunction. All procedures were performed by the three interventional radiologists at our center who had PTCB experience of 11, 15, and 25 years. The following information was recorded from the patient electronic records: age, sex, operator, PTCB approach (left or right approach), BS site, BS length, imaging findings (eccentricity, rat tail, truncation, and filling defect sign), maximum lesion diameter at the BS area, and final pathological results.

### Procedures

Routine blood testing including liver function, kidney function, electrolytes, coagulation function, and tumor markers as well an electrocardiogram were examined before the procedure. Additionally, both non-contrast and enhanced upper abdomen CT or MR were performed to understand the dilatation of the bile duct, the location of BS, the tumor size, and the planned puncture path.

The patient was laid supine on the DSA (Artis zeego, Siemens, Germany) examination bed, and a mixed solution of dizosin (10 mg) and dexmedetomidine hydrochloride (400 µg) was injected intravenously to obtain a satisfactory analgesic state. After routine disinfection and towel draping at the right costal area, local anesthesia was performed with 2% lidocaine (5 mL) at the skin puncture point, which was determined by preoperative enhanced CT/MR or intraoperative US. A 21-G Chiba needle (Cook, USA) was used to puncture the dilated bile duct, a platinum microwire (0.018 in. × 30 cm) was introduced to the biliary system after cholangiography (320 mg iodine/100 mL, Hengrui, Jiangsu, China, diluted 50% for use), a 6-F dilator was introduced along the micro hydrophilic membrane wire, and another cholangiogram was performed to confirm the BS length and extent. A 0.035-in. wire and 5-F KMP catheter were exchanged with a 6-F dilator, and the BS was opened with the cooperation of the guide wire and catheter until the catheter finally entered the duodenum. Another 0.035-in. strength guide wire (145 cm in length, Radiofocus M, Terumo, Tokyo, Japan) was introduced to establish the skin-bile duct-duodenum approach. Along the guide wire, a 9-F short sheath (23 cm long, cordis, USA) was introduced, whose end was located above the BS segment. After confirmation by angiography, the biopsy forceps (6.0 mm in diameter, Nanjing MicroPort, China) were introduced to the BS through a 9-F sheath, the biopsy forceps were opened and pushed forward for 5–10 mm, and the biopsy forceps were then tightened to clamp the tissue. The biopsy forceps were withdrawn, and the tissue was fixed in a 4% formaldehyde solution. PTCB was repeated three to six times until the sample size met the needs of clinical diagnosis (assessed by a pathologist who had more than 15 years of experience). 8.5-F internal and external drainage tubes (Cook, USA) were introduced after biopsy, and the lateral drainage holes were allowed to cross the BS area. Liver protective and anti-infective drugs were given after the operation for 3–7 days.

### Definition

Technical success was defined as successfully obtaining enough biliary tissue to complete the pathology examination. The final diagnosis was confirmed by surgery, other histology, or cytology (i.e., percutaneous fine needle aspiration biopsy and bile cytology). If there was no histology or cytology, the patient was followed up for at least 6 months, and the patient was diagnosed as malignant if the size of the lesions was significantly increased and/or metastasis was found by imaging examination. If there was no obvious disease progression in clinical manifestations or imaging findings, it was diagnosed as benign. Diagnostic indicators included sensitivity, specificity, FNV, and false-positive value (FPV). PTCB-related complications were defined according to the International Society of Interventional Radiology (SIR) operating guidelines [14]. In view of multifactorial analysis, malignant tumors are divided into two groups according to their origin: cholangiocarcinoma (malignant tumors originating from the bile duct epithelium) and non-cholangiocarcinoma tumors (malignant tumors other than cholangiocarcinoma, such as pancreatic cancer, gallbladder cancer, hepatocellular carcinoma, duodenal cancer, and hilar metastatic lymph nodes).

### Statistical analysis

Continuous data are expressed as the means ± standard deviation, and a *t* test was used to compare the indices of alanine aminotransferase, total bilirubin, and direct bilirubin before and 1 week after drainage. Univariate and multivariate logistic regression analyses were used to identify the independent prognostic factors associated with FNV. Statistical analysis was performed using the SPSS software version 21.0 (IBM, Chicago, IL). A *p* value < 0.05 was considered to indicate a significant difference.

## Results

All patients (male:female = 101:93, mean age (61.0 ± 9.3) years old) presented with clinical symptoms of BS, and preoperative enhanced CT/MR showed slight to severe intrahepatic bile duct dilatation. Preoperative total bilirubin (TB), direct bilirubin (DB), and alanine aminotransferase (ALT) were 169.4 ± 58.5 μmol/L (range 93.6–342.1), 135.0 ± 50.4 μmol/L (range 76.4–277.9), and 91.9 ± 40.6 U/L (range 24.4–198.6), respectively, and obtained a sufficient sample for diagnosis, and the technical success rate was 100%. The right and left puncture approaches were performed in 164 and 30 cases, respectively. There were 12 (6.2%), 54 (27.8%), 89 (45.9%), and 39 (20.1%) cases of BS involving the intrahepatobiliary duct (IHD), hilar duct, middle extrahepatic bile duct, and distal extrahepatic bile duct, respectively. The average number of biopsies was 4.0 ± 1.3 times/case. The final diagnoses were 176 cases of malignant tumors, including 123 cases of cholangiocarcinoma and 53 cases of non-cholangiocarcinoma tumors (24 cases of pancreatic cancer, 9 cases of gallbladder cancer, and 20 cases of metastatic cancers (gastric cancer, liver cancer, lymphoma, intestinal cancer metastasis)). The final diagnosis of malignant disease was made by surgical pathological examination (*n* = 29), fine needle aspiration biopsy (*n* = 23), and cytological evidence of bile and ascites (*n* = 11). The imaging indices of regular follow-up showed that the local lesions were enlarged or metastasized (*n* = 113). Eighteen cases were diagnosed as benign stenosis, of which 5 were confirmed by surgical pathology. In the absence of surgical pathology results, 13 benign diseases were based on a medical follow-up of at least 6 months, and neither the clinical course nor imaging examination showed evidence of disease progression (Table 1).

**Table 1:** Results of PTCB and final diagnosis

PTCB	Final diagnosis		Total
	Positive result	Negative result	
Positive result	144	0	144
Negative result	32	18	50
Total	176	18	194

Thirty-two cases were identified as false-negative results, including chronic inflammation (*n* = 21) and fibrous connective tissue (*n* = 11). Among them, 8 cases subsequently obtained malignant pathological evidence following secondary PTCB, and the remaining 24 were diagnosed as false-negative by medical imaging follow-up. The results of this study showed that the sensitivity, specificity, false-positive value, and FNV of PTCB were 81.8%, 100%, 0%, and 18.2%, respectively. The overall diagnostic accuracy of PTCB for all biliary strictures was 74.2% (144 of 194 cases were confirmed) (Table 2). TB decreased from 169.4 ± 58.5 μmol/L before drainage to 82.4 ± 32.2 μmol/L at 1 week, which was a significant difference (*t* = 9.3, *p* = 0.00). DB decreased from 135.0 ± 50.4 μmol/L before drainage to 61.3 ± 22.5 μmol/L at 1 week, which was also a significant decrease (*t* = 9.5, *p* = 0.00). Alanine aminotransferase decreased from 91.9 ± 40.6 U/L before drainage to 55.3 ± 17.8 U/L at 1 week, which was significant (*t* = 5.1, *p* = 0.00).

**Table 2:** Baseline characteristics and univariate risk factor analysis of 194 patients with false-negative results

Influence factor	All patients (*n* = 194)	False negative (*n* = 32)	Univariate analysis, OR (95% CI)	*p* value
Age (years)	61 ± 9.29	63 ± 9.09	1.02 (0.98–1.06)	0.36
Male (female)	101 (93)	16 (16)	1.10 (0.52–2.36)	0.80
Operator				
Operator 1	83	12	1	
Operator 2	80	5	1.37 (0.60–3.13)	0.46
Operator 3	31	15	1.14 (0.37–3.54)	0.82
Puncture approach				
Right side	164	26	1	
Left side	30	6	1.33 (0.50–3.56)	0.58
Forcep times	4.0 ± 1.28	4.0 ± 1.10	1.12 (0.84–1.50)	0.45
BS part				
Intrahepatic BS	12	8	1	
Hilar BS	54	2	0.87 (0.16–4.73)	0.87
MEBS	89	15	1.01 (0.20–5.10)	0.99
DEBS	39	7	1.09 (0.20–6.14)	0.92
The length of BS	2.9 ± 0.72	2.7 ± 0.80	1.30 (0.77–2.18)	0.33
Imaging features				
Eccentricity sigh	69	5	1	
Truncation sign	48	7	0.42 (0.14–1.24)	0.12
Rat tail sign	55	15	0.53 (0.20–1.40)	0.20
Filling defect sign	22	5	1.06 (0.34–3.34)	0.92
Max. diameter at BS part	2.5 ± 1.38	3.3 ± 1.28	1.56 (1.21–2.01)	0.00*
Tumor type				
Cholangiocarcinoma	123	19	1	
Non-cholangiocarcinoma tumors	53	13	4.56 (2.04–10.19)	0.00*
Benign BS	18	0	0.00	1.0

*OR* odds ratio, *BS* biliary stricture, *MEBS* middle extrahepatic biliary stricture, *DEBS* distal extrahepatic biliary stricture

^*^p < 0.05

Thirteen cases (6.7%) experienced early complications, including nine cases of minor biliary bleeding (total volume ≤ 10 mL), all of which were transient and gradually decreased and disappeared within 2–72 h. Four patients experienced acute pancreatitis (abdominal pain with continuous elevation of blood amylase) and recovered after 3–7 days of symptomatic treatment. No bile duct perforation, hepatic artery pseudoaneurysm, or severe bleeding was found in the study.

Univariate logistic analysis was used to determine the factors predictive for FNV, including age, sex, operator, biopsy approach (right or left approach), number of biopsies, location of obstruction, length of obstruction, imaging findings, maximum diameter of tumor, and primary tumor type (cholangiocarcinoma or non-cholangiocarcinoma tumor). Univariate logistic analysis showed that FNV was related to the maximum diameter of the bile duct tumor and the type of primary tumor (Table 2). Multivariate logistic regression analysis showed that only non-cholangiocarcinoma tumors (odds ratio 7.5, 95% CI (1.7–32.6), *p* = 0.007) were independent prognostic factors for FNV (Table 3).

**Table 3:** Multivariate analysis of false-negative results

Influence factor	Multivariate analysis, OR (95% CI)	*p* value
Max. diameter at BS part	0.835 (0.510–1.367)	0.47
Imaging features		
Eccentricity sigh	1	
Truncation sign	0.347 (0.109–1.104)	0.07
Rat tail sign	0.503 (0.178–1.421)	0.20
Filling defect sign	1.086 (0.309–3.817)	0.90
Tumor type		
Cholangiocarcinoma	1	
Non-cholangiocarcinoma tumors	7.501 (1.724–32.630)	0.01*
Benign	0	1.00

*OR* odds ratio, *BS* biliary stricture

^*^p < 0.05

## Discussion

**Fig. 1:**
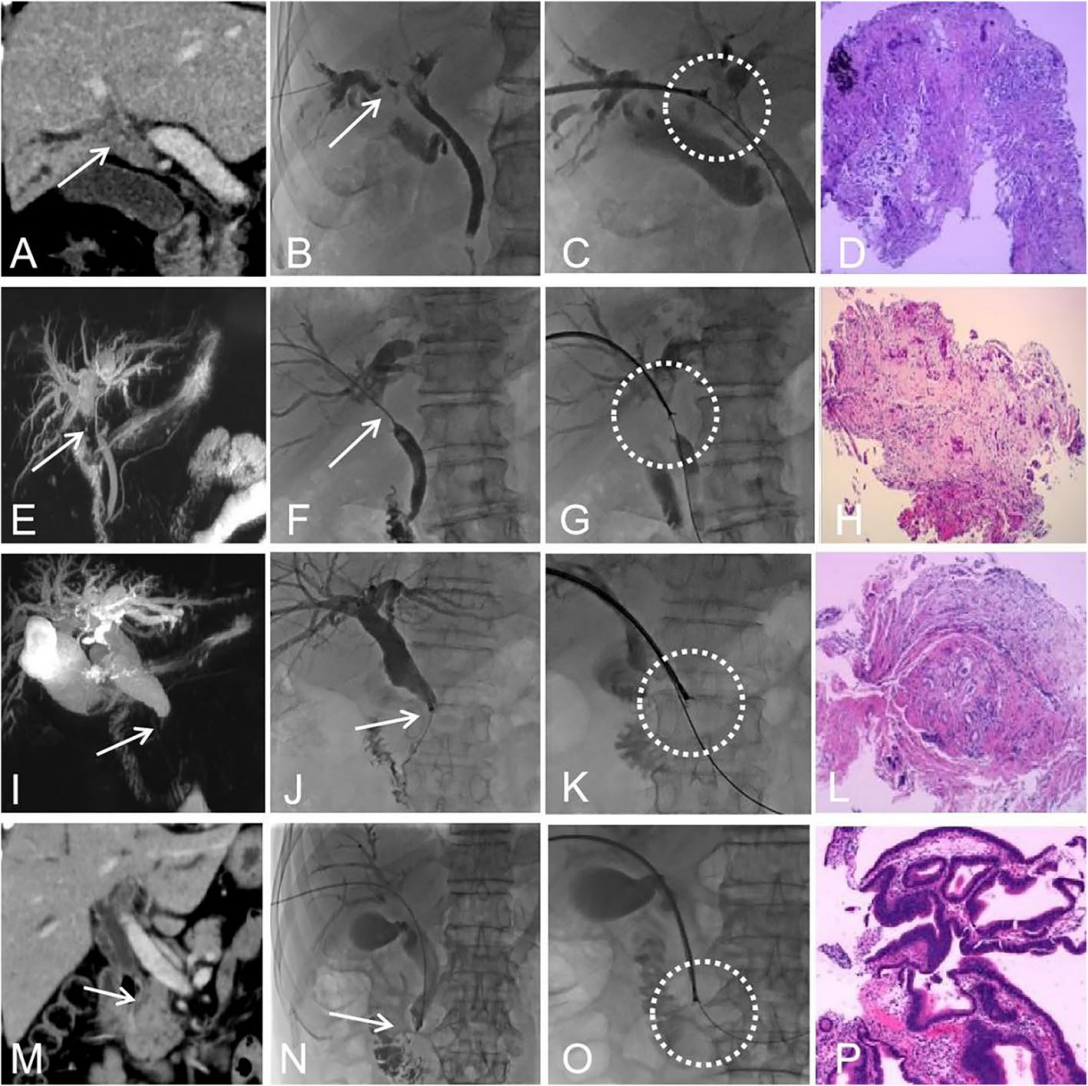
PTCB for four biliary different sites. Case 1 (**A**–**D**), a 59-year-old male, had intrahepatic bile duct obstruction with jaundice. Preoperative enhanced CT coronal view (**A**) showed intraluminal tumor (arrow) and distal bile duct dilatation; percutaneous cholangiography showed intrahepatic filling defect sign in the hilar and left and right biliary ducts (**B**, arrow). The biopsy forceps were introduced through the 9F sheath, and the PTCB was completed on the obstruction site (**C**, circle). The pathological result showed cholangiocarcinoma (**D**). Case 2 (**E**–**H**), a 71-year-old female, had hilar bile duct obstruction. Preoperative MRCP suggested hilar bile duct obstruction (**E**, arrow) with obvious dilatation of the intrahepatic bile duct; percutaneous cholangiography showed significant stricture of the hilar bile duct (**F**, arrow), and the position was consistent with preoperative MRCP. The biopsy forceps were introduced through the 9F sheath, and PTCB was completed on the obstruction site (**G**, circle); the pathological results showed cholangiocarcinoma (**H**). Case 3 (**I**–**L**), a 54-year-old male, had middle extrahepatic bile duct obstruction. Preoperative MRCP (**I**, arrow) showed extrahepatic middle bile duct obstruction and severe dilatation of the bile duct above the obstruction. Percutaneous cholangiography showed severe obstruction in the middle segment of the bile duct, which coincided with the location of preoperative MRCP (**J**, arrow). The biopsy forceps were introduced through the 9F sheath, and PTCB was completed on the obstruction site (**K**, circle); pathological results showed cholangiocarcinoma (**L**). Case 4 (**M**–**P**), a 65-year-old male, had low biliary obstruction. The coronal position of preoperative enhanced CT (**M**) showed low obstruction of the extrahepatic common bile duct (arrow) and severe dilatation of the bile duct above the obstruction. Percutaneous cholangiography showed severe obstruction of the lower bile duct, which was consistent with the position shown by preoperative enhanced CT (**N**, arrow). The biopsy forceps were introduced through the 9F sheath, and the PTCB was completed on the obstruction site (**O**, circle). The pathological result showed pancreatic cancer (**P**)

BS caused by malignant tumors in and around the bile duct have no specific clinical manifestations at an early stage, and more than 70% of cases are not surgical candidates at diagnosis [15]. A recent meta-analysis showed that the overall survival for cholangiocarcinoma was less than 15% with extremely poor outcomes [16]. Cholangiocarcinomas are small when BS occurs, and US, CT, or MR can show the anatomical relationship of the lesions but cannot determine the histopathological diagnosis. Since 2000, our center has developed a technique to address this limitation: the biliary obstruction was opened by a catheter and guidewire during percutaneous transhepatic cholangial drainage; then, the skin-bile duct-duodenal approach was established by a strengthened guidewire, and a 9-F short sheath instead of endoscope was advanced to establish a PTCB channel to complete the forceps biopsy [17]. This retrospective study showed that the sensitivity and specificity were 81.8% and 100%, respectively, which was equivalent to the diagnosis and treatment efficiency reported by other centers (70–93%) [6, 18–20] and higher than the results of simple cell brush examination (35–60%) [21–23]. The reason may be that PTCB can obtain more deep-level tissue structure than surface tissue compared with brush examination (Fig. 1). Moreover, the internal environment of cholangiocarcinoma is rich in fibrous connective tissue, and it is difficult to obtain enough cytological evidence by brush examination. ERCP-guided biopsy of intrahepatic obstruction is more suitable for low-level obstruction of the bile duct, and the diagnostic efficiency is 54–82% [24–26]. However, hilar and intrahepatic BS is still technologically difficult for inexperienced operators. More importantly, ERCP requires general anesthesia, which increases the surgical cost; PTCB can be performed under local anesthesia, which is more suitable for elderly patients with cardiopulmonary dysfunction. It has been reported that intraductal ultrasound (IDU)-assisted ERCP-guided biopsy can improve the diagnostic efficiency [27], but IDU is expensive, and the majority of hospitals in China do not have such equipment. Therefore, regardless of the requirements for subjectivity and objectivity, PTCB has a wider range of indications and a higher cost efficiency. Theoretically, the risk of intraluminal biopsy of the bile duct wall includes damage to the blood vessels in the adjacent Gleason system or bile leakage. Our study found no such serious complications, which emphasizes the safety of this technology.

**Fig. 2:**
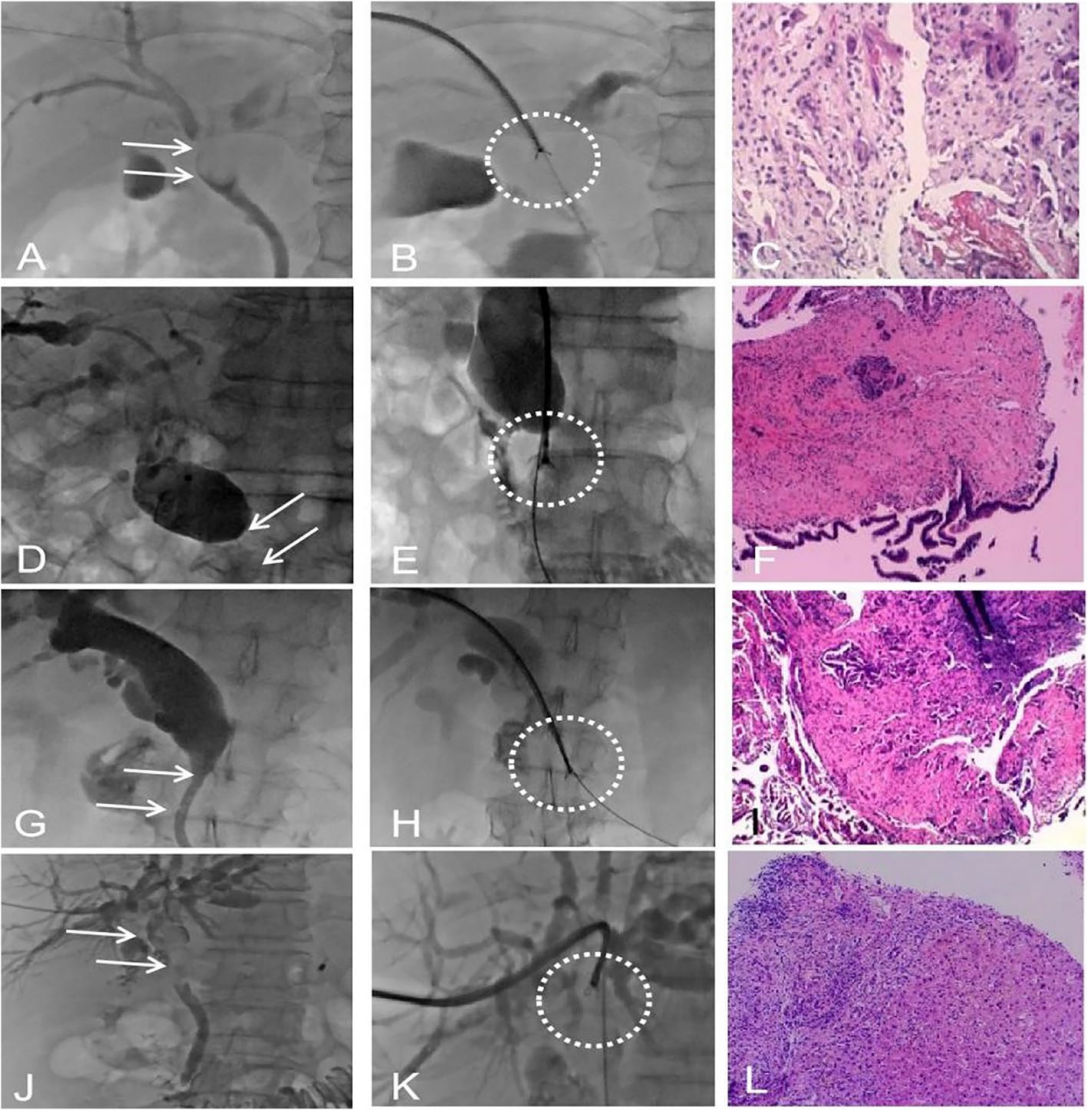
PTCB for four different imaging features. Case 1 (**A**–**C**), a 46-year-old male, had metastatic carcinoma of the hepatic hilum, and preoperative fluoroscopy showed the biliary eccentricity sign (**A**, arrow). The biopsy forceps were introduced through the 9F sheath, and the PTCB was completed on the obstruction site (**B**, circle). The pathological result was hilar adenocarcinoma (**C**). Case 2 (**D**–**F**), a 52-year-old female, had a malignant obstruction in the middle segment of the bile duct, and preoperative fluoroscopy showed a local truncation sign (**D**, arrow). The biopsy forceps were introduced through the 9F sheath, and the PTCB was completed on the obstruction site (**E**, circle). The pathological result was cholangiocarcinoma (**F**). Case 3 (**G**–**I**), a 76-year-old male, had a lower part of the biliary obstruction. Preoperative cholangiography showed low obstruction with the rat tail sign (**G**, arrow). The biopsy forceps were introduced through the 9F sheath, the PTCB was completed on the obstruction site (**H**, arrow), and the pathological result was cholangiocarcinoma (**I**). Case 4 (**J**–**L**), a 50-year-old female, had a hilar obstruction. Cholangiography showed an extensive filling defect sign of the bile duct (**J**, arrow). The biopsy forceps were introduced through the 9F sheath, and the PTCB was completed on the obstruction site (**K**, circle). The pathological result was cholangiocarcinoma (**L**)

In this study, imaging features under fluoroscopy were included in the analysis of the FNV of PTCB for the first time, which is a major characteristic of this study (Fig. 2). The rat tail, truncation, filling defect, and eccentricity sign were 28.4%, 24.7%, 11.3%, and 35.6%, respectively, but there were no statistically significant differences using univariate and multivariate multifactor analyses. It is posited that: (1) One sign, e.g., truncation sign, can have multiple causes, including cholangiocarcinoma, metastatic cancer, pancreatic cancer, or benign lesions. (2) The same disease can have multiple signs. For example, cholangiocarcinoma can show centripetal or eccentric stenosis and can also show truncation signs or mouse tail signs. (3) The same anatomical site can have a BS caused by any of multiple diseases, e.g., the hilar part can be cholangiocarcinoma, liver cancer, or metastatic cancer. (4) The signs are not typical. Strictures can be caused by metastatic and pancreatic cancers directly compressing or invading the bile duct; gallbladder cancer can have direct invasion, lymph node metastasis, or intraluminal dissemination of the bile duct; and gastrointestinal tumors can metastasize to the hilar or hepatoduodenal ligament to cause lymph node enlargement and compression or infiltration of the bile duct. Theoretically, there should be obvious signs of pushing and displacement of the bile duct wall. However, in practice, this sign is very rare, mainly because these non-cholangiocarcinoma tumors still exhibit infiltrative growth. Most of them directly invade the surrounding tissue space and lymphatic or blood vessels, infiltrating and destroying the bile duct wall and its surrounding tissue structure.

Univariate analysis showed that the FNV was related to tumor size, but multivariate analysis showed no significant difference. It is not difficult to understand that PTCB is different from percutaneous puncture and cutting biopsy. The biopsy site of the intraluminal PTCB is the most stenotic site of the BS lumen, indicating abundant tumor-infiltrating tissue. Sato et al. [28] demonstrated that the biopsy specimen was only taken from the superficial part of the mucosa and fibromuscular layer of the bile duct. The space of the bile duct was small, and the malignant proliferation of the bile duct epithelium led to local cancer cell aggregation. Therefore, more cancerous tissues could be obtained by clamping the BS area. As a result, PTCB is less helpful in detecting exogenous tumors or tumors deep in the bile duct wall. A study from Terasaki et al. demonstrated that the results of PTCB for bile duct obstruction caused by metastatic disease compression largely depend on the depth and degree of invasion of the external tumor to the bile duct wall [29].

Park et al. [30] analyzed the data of 271 cases of bile duct PTCB and showed that, in addition to the tumor type, the lesions in the periampullary segment of the common bile duct were also independent risk factors for FNV. They explained that the clamp is always clamped to the sidewall of the common bile duct rather than the BS because the bile duct in the ampullary region was angled. However, our study showed that tumor location was not a significant factor. This may be related to the use of instruments and biopsy methods: (1) The 9-F sheath was bent by 60% with external force before PTCB to adapt to the hilar curvature. (2) The sheath used in this center is thicker than that used in other centers and can therefore accommodate large biopsy forceps and take sufficient samples. Wu et al. [31] showed that large biopsy forceps can obtain more samples (6.0 mm vs. 4.5 mm) to improve the accuracy rate. (3) Our center requires that the sheath be placed above the BS to touch the lesion as closely as possible. A recent large-scale study including 241 patients with BS showed that stenosis length ≥ 30 mm was an important indicator for positive diagnosis with PTCB [32]. However, in our study, stenosis length was not associated with FNV. This may be related to the sampling method. It is recommended to adopt segmental sampling (sampling at the low-middle and upper parts to obtain more representative samples) for long BS. Theoretically, the number of biopsies should be of great significance for FPV, but univariate and multivariate analyses showed that the number of biopsies was not related. Considering that most of the specimens in the bile duct are adenocarcinoma, as long as the most serious part of BS, i.e., the most serious part of tumor invasion, can be obtained, the materials should be representative; the average number of clamps at our center was 4, while other studies reported 3 [30]. A sufficient sample size can be obtained by increasing the number of biopsies.

This retrospective study has certain limitations: (1) Only 14.9% of the cases were confirmed by final surgery and pathology, while up to 62.9% of the patients were only confirmed by imaging follow-up. (2) The follow-up time was at least 6 months, which is still short given some bile duct tumors show inertia. (3) Tumor response to therapy (chemotherapy or irradiation) was confounding to the final follow-up at 6 months as done in this study. (4) This study is only a single-center study, which limited its statistical power to a certain extent. (5) Only one pathologist evaluated the samples from the biopsy site, which can lead to a decrease in reliability. In conclusion, PTCB is safe and reliable in the diagnosis of malignant biliary obstruction with few complications. Non-cholangiocarcinoma tumors are an independent risk factor for FNV.

## Data Availability

The datasets used or analyzed during the current study are available from the corresponding author upon reasonable request.

## Abbreviations

BS: Biliary stricture
FNV: False-negative value
FPV: False-positive value
PTCB: Percutaneous transluminal clamp biopsy
